# Species delimitation based on mtDNA genes suggests the occurrence of new species of *Mesocestoides* in the Mediterranean region

**DOI:** 10.1186/s13071-018-3185-x

**Published:** 2018-12-04

**Authors:** Antonio Varcasia, Daria Sanna, Marco Casu, Samia Lahmar, Giorgia Dessì, Anna Paola Pipia, Claudia Tamponi, Gabriella Gaglio, Gabriela Hrčková, Domenico Otranto, Antonio Scala

**Affiliations:** 1 Dipartimento di Medicina Veterinaria, Università di Sassari, Sassari, Italy; 2 Dipartimento di Scienze Biomediche, Università di Sassari, Sassari, Italy; 3 National School of Veterinary Medicine, Laboratory of Parasitology, Sidi Thabet, Tunisia; 4 Dipartimento di Scienze Veterinarie, Università di Messina, Messina, Italy; 5 Institute of Parasitology, Slovak Academy of Sciences, Košice, Slovak Republic; 6 Dipartimento di Medicina Veterinaria, Università di Bari, Bari, Italy

**Keywords:** *Mesocestoides*, Dog, Cat, Fox, Genetic structuring, Species delimitation, Mediterranean region

## Abstract

**Background:**

This study is the first contribution to the molecular taxonomy of *Mesocestoides* spp. from domestic and wild carnivores in the Mediterranean area. A total of 13 adult worms and 13 larval stages of *Mesocestoides* spp. were collected from domestic and wild carnivore hosts in Italy and Tunisia. Samples collected in the Slovak Republic were used as comparative samples from outside the Mediterranean. The genes cytochrome *c* oxidase subunit 1 (*cox*1) and NADH dehydrogenase subunit 1 (*nad*1) of the mitochondrial genome were used as molecular markers to investigate the presence of cryptic *Mesocestoides* species in the area analysed.

**Results:**

Results were consistent in showing three well-supported clusters of *Mesocestoides* spp. in southern Italy and Tunisia, which were strongly divergent from *Mesocestoides litteratus*, *M. corti* and *M. lineatus.* High levels of genetic variation and no evidence of geographical structuring was found between the clusters.

**Conclusions:**

Considering the low dispersal capability of the intermediate hosts of *Mesocestoides* spp., the lack of geographical structuring among the Mediterranean regions could be due to a high potential for dispersion of the definitive hosts. This study provides a foundation for future formal descriptions of new species of the genus *Mesocestoides* in the Mediterranean area.

**Electronic supplementary material:**

The online version of this article (10.1186/s13071-018-3185-x) contains supplementary material, which is available to authorized users.

## Background

The genus *Mesocestoides* (Cyclophyllidea, Mesocestoididae) includes parasites with unique peculiarities in many aspects of their biology, which have yet to be revealed [1]. Two intermediate hosts are likely required for the completion of the *Mesocestoides* life-cycle [2], with the first larval stage developing in coprophagous arthropods, and the second (i.e. tetrathyridium) in a wide variety of hosts (e.g. rodents, amphibians, reptiles and birds) [1, 3, 4]. Adult *Mesocestoides* worms have been recorded in up to 13.8% of cats, 26.5% of dogs, 70% of jackals and 81.8% of foxes [2, 5–8]. The latter species seem to be the most important definitive hosts for these parasites as confirmed by a recent paper that highlighted a prevalence of 84.1% in red foxes from Poland [9]. Wild and domestic carnivores could also serve as second intermediate hosts [10] since tetrathyridium larvae can multiply asexually by longitudinal fission, penetrate the intestinal wall, invade the peritoneal cavity of the hosts and eventually cause life-threatening peritonitis [11–13]. In addition, *Mesocestoides* spp. are potentially zoonotic, being reported in human infections (at least 27 cases) following the consumption of raw or undercooked snake, chicken and wild game viscera [14, 15].

The distribution of *Mesocestoides* species is not well delineated due to their high degree of phenotypic plasticity, which hinders a clear morphological delineation of the species [5]. Furthermore, identification at the species level is not possible for the larval stages from intermediate hosts [16] or also when parasites are recovered incomplete, as gravid proglottids. This might have led to the failure of correct species identification in the past [5]. Seven species of *Mesocestoides* have been recorded in Europe (in the Czech Republic, Slovak Republic and Spain) [4, 5, 16, 17] with *M. litteratus* and *M. lineatus* being the most widely distributed species. Although adults of *Mesocestoides lineatus* and *Mesocestoides litteratus* may be differentiated morphologically by subtle differences in the structure of the cirrus-sac, the number of testes and the position of the ovary and vitellarium [18, 19], a biomolecular confirmation of the morphological diagnosis is not exhaustive [20–25]. To date, information regarding intermediate and paratenic hosts of *M. litteratus* and *M. lineatus* in natural conditions is lacking [4].

Recently, a clear genetic distinction of *M. lineatus* and *M. litteratus* has been investigated in specimens displaying minor differences in male and female reproductive organs of worms collected from red foxes in Slovak Republic [5]. Although these parasites are common in the Mediterranean region [2, 6, 13, 26, 27], very few studies have investigated the taxonomy and the molecular characterization of *Mesocestoides* in this area.

In the present study, several individuals of *Mesocestoides* spp. from different hosts in southern Italy and Tunisia have been studied by sequencing of the cytochrome *c* oxidase subunit 1 (*cox*1) and NADH dehydrogenase subunit 1 (*nad*1) mitochondrial genes, in order to shed new light on the possible occurrence of new genetic variants and/or species. Molecular species delimitation methods were therefore applied. Furthermore, *cox*1 and *nad*1 sequences from *Mesocestoides litteratus* and *M. lineatus* specimens collected in Slovak Republic and deposited in the Parasitic Worms Collection at the Natural History Museum, London, were obtained in this study to be used as comparative material.

## Methods

**Fig. 1:**
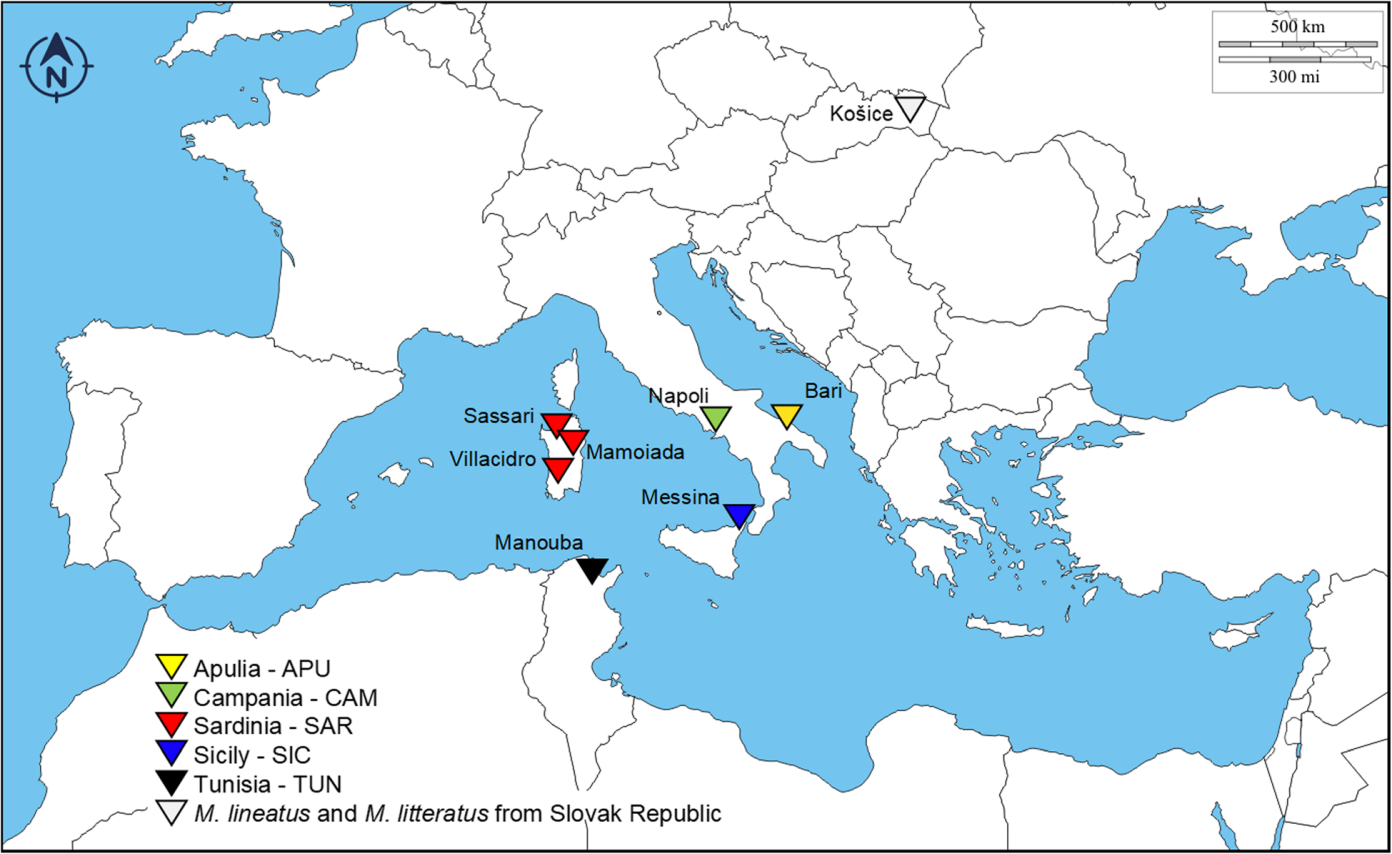
Map of the Mediterranean indicating the sampling sites

The study was carried out on a total of 13 adult worms and 13 larval stages (tetrathyridia) of *Mesocestoides* spp. These were collected between 2014 and 2017 from animals (dogs and cats) referred for clinical visits and elective surgeries or recovered during necropsy (dogs and foxes) at the Veterinary Teaching Hospitals of the Universities of Sassari, Bari, Messina and Naples (Italy), and at the National School of Veterinary Medicine, Sidi Thabet (Tunisia). Details on hosts, parasites and sampling locations are reported in Table 1 and in Fig. 1. Morphological identification of parasites to the genus level was performed, when possible, according to available keys [5]. Fragments from five adult individuals of *M. litteratus* and one of *M. lineatus* found in foxes from Slovak Republic were also included in the study [5] in order to perform phylogenetic analysis**.** These specimens were identified according to Skrjabin [18], mounted on slides and deposited in the Parasitic Worms Collection at the Natural History Museum, London under the accession numbers BMNH 2011.2.2.1-3 and BMNH 2011.2.2.19-20, and have been used as comparative/reference material, in this study focused on parasites of the Mediterranean region.

**Table 1:** Data collection and list of the specimens and the sequences included in the analyses

Sample ID	Host	Parasite stage	Isolated from	Origin	Morphological ID	Sampling site	Region	Country	GenBank ID	
									*cox* *1*	*nad* *1*
APU01	Dog	Tetrathyridium	Peritoneum	Surgery	*Mesocestoides* sp.	Bari	Apulia	Italy	MH463494	MH463520
APU02	Dog	Tetrathyridium	Peritoneum	Surgery	*Mesocestoides* sp.	Bari	Apulia	Italy	MH463495	MH463521
APU04	Dog	Tetrathyridium	Peritoneum	Surgery	*Mesocestoides* sp.	Bari	Apulia	Italy	MH463496	MH463522
APU05	Dog	Tetrathyridium	Liver	Surgery	*Mesocestoides* sp.	Bari	Apulia	Italy	MH463497	MH463523
APU06	Dog	Tetrathyridium	Intestine	Faecal sample	*Mesocestoides* sp.	Bari	Apulia	Italy	MH463498	MH463524
APU07	Dog	Tetrathyridium	Peritoneum	Surgery	*Mesocestoides* sp.	Bari	Apulia	Italy	MH463499	MH463525
APU08	Dog	Tetrathyridium	Peritoneum	Surgery	*Mesocestoides* sp.	Bari	Apulia	Italy	MH463500	MH463526
APU09	Dog	Tetrathyridium	Peritoneum	Surgery	*Mesocestoides* sp.	Bari	Apulia	Italy	MH463501	MH463527
CAM01	Dog	Tetrathyridium	Peritoneum	Surgery	*Mesocestoides* sp.	Napoli	Campania	Italy	MH463505	MH463530
SAR01	Cat	Adult worm	Intestine	Faecal sample	*Mesocestoides* sp.	Villacidro	Sardinia	Italy	MH463502	na
SAR02	Dog	Tetrathyridium	Peritoneum	Surgery	*Mesocestoides* sp.	Sassari	Sardinia	Italy	MH463503	MH463528
SAR03	Fox	Adult worm	Intestine	Necropsy	*Mesocestoides* sp.	Mamoiada	Sardinia	Italy	MH463504	MH463529
SIC01	Cat	Tetrathyridium	Peritoneum	Surgery	*Mesocestoides* sp.	Messina	Sicily	Italy	MH463491	MH463517
SIC02	Dog	Tetrathyridium	Peritoneum	Surgery	*Mesocestoides* sp.	Messina	Sicily	Italy	MH463492	MH463518
SIC03	Cat	Tetrathyridium	Peritoneum	Surgery	*Mesocestoides* sp.	Messina	Sicily	Italy	MH463493	MH463519
TUN01	Dog	Adult worm	Intestine	Necropsy	*Mesocestoides* spp.	Sidi Thabet	Sidi Thabet	Tunisia	MH463506	na
TUN02	Dog	Adult worm	Intestine	Necropsy	*Mesocestoides* spp.	Sidi Thabet	Sidi Thabet	Tunisia	MH463507	MH463531
TUN03	Dog	Adult worm	Intestine	Necropsy	*Mesocestoides* spp.	Sidi Thabet	Sidi Thabet	Tunisia	MH463508	MH463532
TUN04	Dog	Adult worm	Intestine	Necropsy	*Mesocestoides* spp.	Sidi Thabet	Sidi Thabet	Tunisia	MH463509	MH463533
TUN05	Dog	Adult worm	Intestine	Necropsy	*Mesocestoides* spp.	Sidi Thabet	Sidi Thabet	Tunisia	MH463510	na
TUN06	Dog	Adult worm	Intestine	Necropsy	*Mesocestoides* spp.	Sidi Thabet	Sidi Thabet	Tunisia	MH463511	na
SR01^a^	Fox	Adult worm	Intestine	Necropsy	*Mesocestoides litteratus*	Košice	Košice	Slovak Republic	MH463512	MH463534
SR02^a^	Fox	Adult worm	Intestine	Necropsy	*Mesocestoides litteratus*	Košice	Košice	Slovak Republic	MH463513	MH463535
SR03^a^	Fox	Adult worm	Intestine	Necropsy	*Mesocestoides litteratus*	Košice	Košice	Slovak Republic	MH463514	MH463536
SR04^a^	Fox	Adult worm	Intestine	Necropsy	*Mesocestoides lineatus*	Košice	Košice	Slovak Republic	MH463515	MH463537
SR05^a^	Fox	Adult worm	Intestine	Necropsy	*Mesocestoides lineatus*	Košice	Košice	Slovak Republic	MH463516	na

^a^Deposited in the Parasitic Worms Collection at the Natural History Museum, London under the accession numbers BMNH 2011.2.2.1-3 and BMNH 2011.2.2.19-20.

*Abbreviation*: *na* not available as not amplified

DNA was extracted using a commercial PureLink® Genomic DNA Mini Kit (Invitrogen, Carlsbad, California, USA) according to manufacturer’s instructions. Partial fragments of the mitochondrial *cox*1 and *nad*1 genes were amplified by polymerase chain reaction (PCR) following previously described protocols [5, 28–30]. PCR products were purified using a Nucleospin Gel and PCR Clean Up kit (Macherey-Nagel, Düren, Germany) and sent to an external sequencing service (Eurofins Genomics, Ebersberg bei München, Germany). Sequence alignments were performed using BioEdit 7.2.5 [31] and deposited in the GenBank database under the accession numbers MH463491-MH463537 (see Table 1 and Additional file 1: Table S1 and Additional file 2: Table S2 for details). The levels of genetic polymorphism within parasites from the Mediterranean region were assessed using DnaSP 5.10 [32]. A median-joining network [33] was constructed using Network 5.0.0.3 (www.fluxus-engineering.com) to infer the genetic relationships among the haplotypes. A 95% statistical parsimony network analysis was performed using TCS 1.21 [34], aimed at searching for possible disconnections between groups of individuals. The occurrence of genetic structure among samples was investigated by the Bayesian model-based clustering algorithm implemented in Baps 6 [35]. Each analysis was performed 10 times with a vector of values (1–10) for K each with 5 replicates.

For the phylogenetic analysis, two enlarged datasets for both *cox*1 and *nad*1 markers were built by aligning the sequences obtained in the present study with all comparable sequences available on GenBank for *M. litteratus*, *M. lineatus* and *M. corti/M. vogae* [5, 36, 37] (see Figs. 4 and 5 for details). Two sequences from Italy (Tuscany, GenBank: JQ740884; and Sicily, GenBank: KU821650) attributed to *Mesocestoides* spp. were also included in the *cox*1 dataset. A sequence of *Echinococcus multilocularis* from GenBank was used as the outgroup (Figs. 4, 5 and Table 1). The *cox*1 gene dataset included 26 sequences obtained in the present study (see Table 1 for details) and 40 from GenBank. For the *nad*1 gene, the fragment analysed for phylogeny was restricted to 202 bp in order to obtain an overlapping segment between our sequences and those from GenBank. The new *nad*1 dataset included 21 sequences obtained in the present study (see Table 1 for details) and 25 sequences from GenBank.

In order to test the phylogenetic signal [38] and the adequacy of taxonomic coverage, the likelihood-mapping analysis of 10,000 random quartets was performed using TreePuzzle 5.3 [39, 40]. The datasets were used to plot a phylogenetic tree using the maximum likelihood (ML) algorithm implemented in MEGA7 [41] with 1000 bootstrap replications, and the Kimura 2-parameter (K2P) as a molecular evolutionary model. The nodes of the trees with bootstrap values lower than 50% were considered not well-supported and thus collapsed.

The combined use of two species delimitation methods, the Automatic Barcode Gap Discovery (ABGD) [42] and the Nucleotide Divergence Threshold (NDT) [42], allowed us to make inferences on the occurrence of taxonomic entities by means of two alternative distance models (simple p-distance for ABGD and Kimura (K80) distance for NDT). ABGD was calculated by means of the ABGD online tool (available at http://wwwabi.snv.jussieu.fr/public/abgd/abgdweb.html) with a prior *P* ranging from 0.001 to 0.12, steps = 10 and relative gap width (X) = 1. The NDT method was applied by means of a script written in the R statistical environment (available at https://cran.r-project.org/) and described in [43–45]. Estimates of evolutionary divergence over sequence pairs between groups were conducted in MEGA7 to evaluate the genetic distance between taxa by using the Kimura 2-parameter model.

## Results

The correct taxonomic attribution of specimens from Slovak Republic used as comparative material in the present study, was verified via a BLASTsearch against the available data in the GenBank nucleotide database. They were attributed to *M. litteratus* and *M. lineatus* respectively (see Table 1 for details on species and GenBank accession numbers). The analysis of the *cox*1 dataset evidenced two haplotypes for *M. litteratus* (*n* = 3, S = 2, h = 0.667, π = 0.00660) and one haplotype for *M. lineatus* (*n* = 1). The analysis of the *nad*1 dataset evidenced two haplotypes for *M. litteratus* (*n* = 3, S = 3, h = 0.667, π = 0.00536) and one haplotype for *M. lineatus* (*n* = 2). The phylogenetic analysis below reported for the Mediterranean region corroborated the taxonomic attribution of specimens from Slovak Republic.

Overall, high levels of genetic variation were found for the *cox*1 dataset (373 bp long) among 21 *Mesocestoides* specimens from the Mediterranean region, with rather low indices of genetic divergence found for the samples from Tunisia (h = 0.800, π = 0.024) (see Table 2 for estimates of genetic divergence).

**Table 2:** Sample sizes and genetic diversity estimates obtained for the mitochondrial regions, *cox*1 (378 bp) and *nad*1 (558 bp). Sites with gaps were not considered. Sample codes are listed in Table 1

Sample	n	S	H	h	π
*cox*1					
Apulia	8	37	5	0.857	0.03380
Campania	1	0	1	0.000	0.00000
Sardinia	3	37	3	1.000	0.06613
Sicily	3	42	3	1.000	0.07685
Tunisia	6	15	4	0.800	0.02377
Total	21	63	11	0.914	0.04708
*nad*1					
Apulia	8	24	4	0.750	0.01773
Campania	1	0	1	0.000	0.00000
Sardinia	2	68	2	1.000	0.12186
Sicily	3	78	3	1.000	0.09550
Tunisia	3	3	2	0.667	0.00358
Total	17	82	9	0.897	0.04502

*Abbreviations*: *n* sample size; *S* number of polymorphic sites, *H* number of haplotypes, *h* haplotype diversity, π nucleotide diversity

**Fig. 2:**
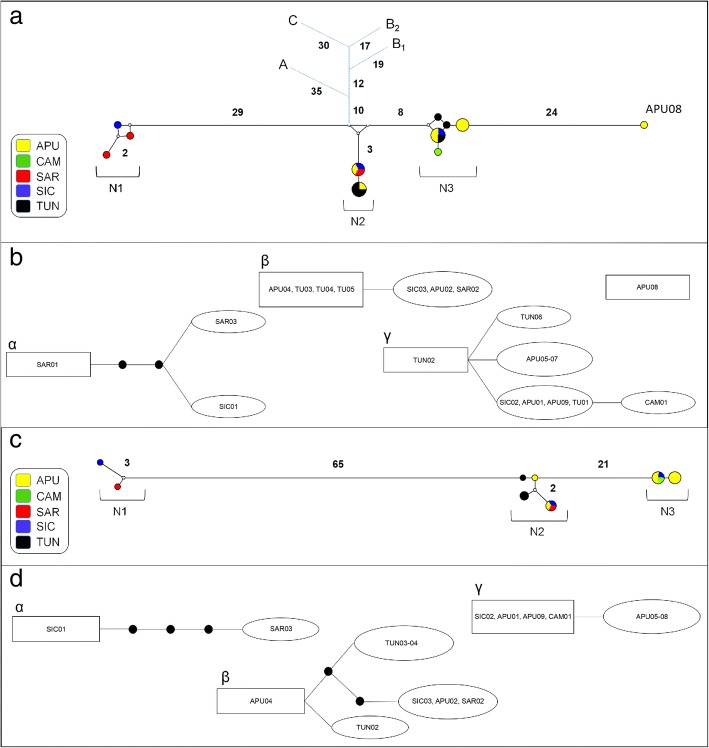
Network analysis. **a**, **b** *cox*1 dataset; **c**, **d** *nad*1 dataset. **a**, **c** Median-joining networks with haplotypes coloured according to their geographical distribution. Small white dots on the nodes show median vectors representing hypothetical connecting sequences, calculated with a maximum parsimony method. The numbers of mutations between haplotypes greater than one are reported on the network branches. In the median-joining networks based on *cox*1 dataset (**a**) the short blue branches represent the connection with the other species. *Abbreviations*: A, *Mesocestoides litteratus*; B_1_ and B_2_, *M. lineatus* from Mongolia and Slovak Republic, respectively; C, *M. corti*. **b**, **d** Clusters retrieved using 95% statistical parsimony networks. The number of mutations greater than one are shown as black dots on the network branches. The haplotype in a square has the largest outgroup weight. *Abbreviations*: APU, Apulia; CAM, Campania; SAR, Sardinia; SIC, Sicily; TUN, Tunisia

**Fig. 3:**
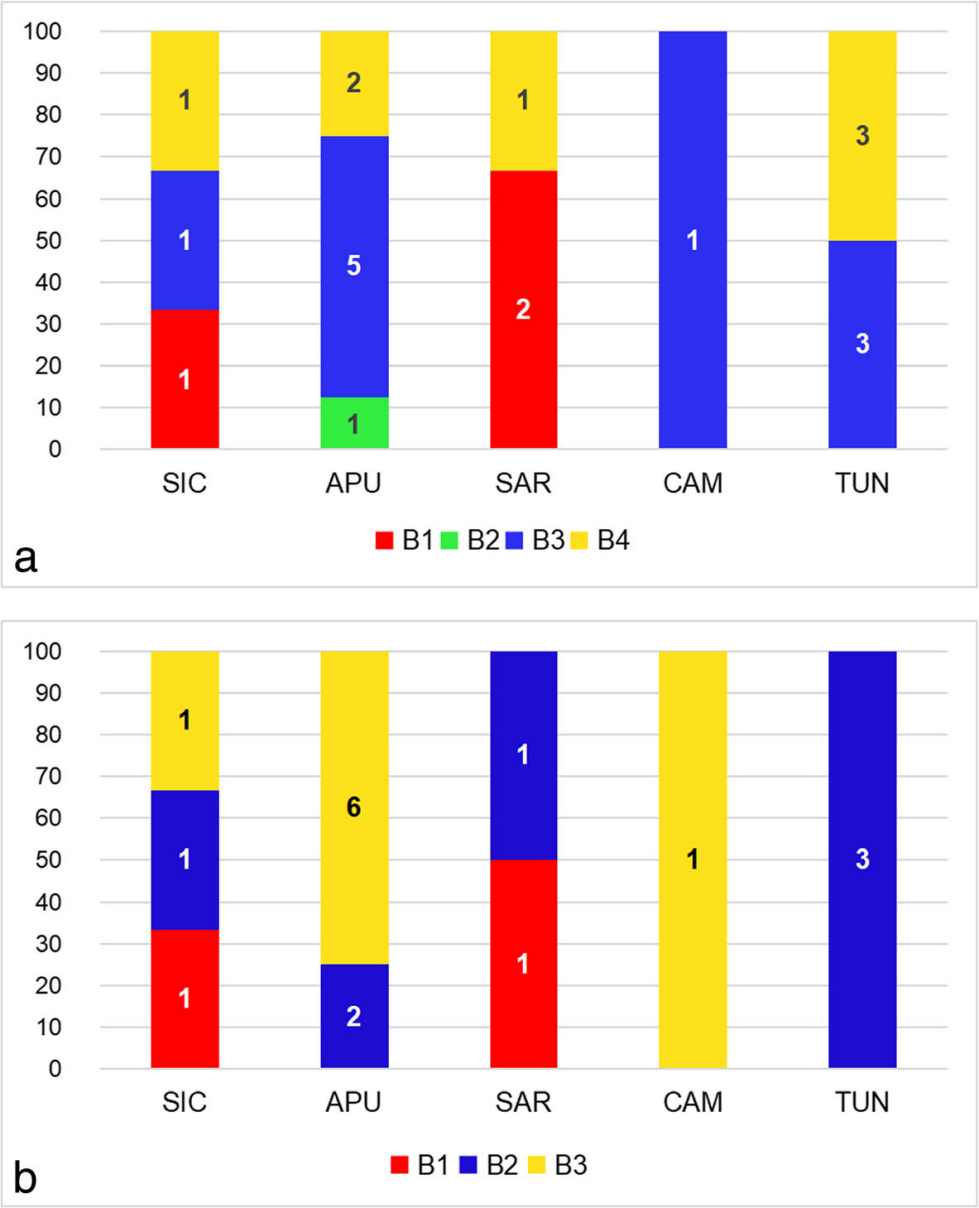
Distribution of the groups identified by Bayesian model-based clustering implemented in Baps 6 within populations. **a** *cox*1 dataset. **b** *nad*1 dataset. X axis: populations; Y axis: relative frequency of distribution (%). *Abbreviations*: APU, Apulia; CAM, Campania; SAR, Sardinia; SIC, Sicily; TUN, Tunisia. The numbers in bars indicate the absolute frequency of distribution. B1, B2, B3 and B4 indicate the groups identified by Bayesian model-based clustering described in the text

Four *cox*1 haplotypes were shared by 67% of the samples while the remaining haplotypes were unique to single individuals (see Additional file 1: Table S1). Median-joining network analysis revealed the occurrence of three main divergent groups of haplotypes (N1, N2 and N3) (see Fig. 2a for details on the geographical distribution of haplotypes). Statistical parsimony network analysis revealed four disconnected clusters within Mediterranean *Mesocestoides* specimens (Fig. 2b). Three of these clusters (α, β and γ) exactly matched the groups of haplotypes in the median-joining network (N1, N2 and N3, respectively). The highest root weight was shown by a haplotype found in Sardinia (SAR01) for cluster α, by haplotypes found in Tunisia and Apulia (TUN03-05, APU04) for cluster β, and by a haplotype found in Tunisia (TUN02) for cluster γ. The Bayesian model-based clustering implemented in Baps 6 identified four distinct groups of haplotypes (B1, B2, B3 and B4) (see Fig. 3a for details on the geographical distribution of groups). B2 was the least frequent group, being only reported for the highly divergent haplotype from Apulia (APU08).

A 555 bp long alignment for the *nad*1 gene included sequences belonging to 17 *Mesocestoides* specimens from Italy and Tunisia (Table 1). As a possible consequence of a limited homology between universal primers used and the annealing region, scorable *nad*1 sequences were obtained for a reduced number of individuals. Overall, high levels of genetic variation were found in the Mediterranean region, which resulted in a total genetic variability similar to that found for the *cox*1 dataset (see Table 2 for details). Three *nad*1 haplotypes were found in 65% of the samples, while the remaining were unique to single individuals (see Additional file 2: Table S2 for more details). The median-joining network analysis revealed the occurrence of three groups of haplotypes (N1, N2 and N3) (see Fig. 2c for details) almost corresponding to those found for the *cox*1 median-joining network analysis (see Fig. 1a). Accordingly, the statistical parsimony network analysis highlighted the occurrence of three disconnected clusters (α, β and γ) within Mediterranean *Mesocestoides* spp. (see Fig. 2d). The highest root weight was shown by a haplotype found in Sicily (SIC01) for cluster α; by a haplotype found in Apulia (APU04) for cluster β; and by haplotypes found in Apulia (APU01, APU09), Campania (CAM01) and Sicily (SIC02) for cluster γ.

The Bayesian model-based clustering implemented in Baps 6 (see Fig. 3b for details) identified three groups of haplotypes (B1, B2 and B3) which were consistent with three (B1, B3 and B4) of the four groups reported for the *cox*1 Bayesian analysis.

**Fig. 4:**
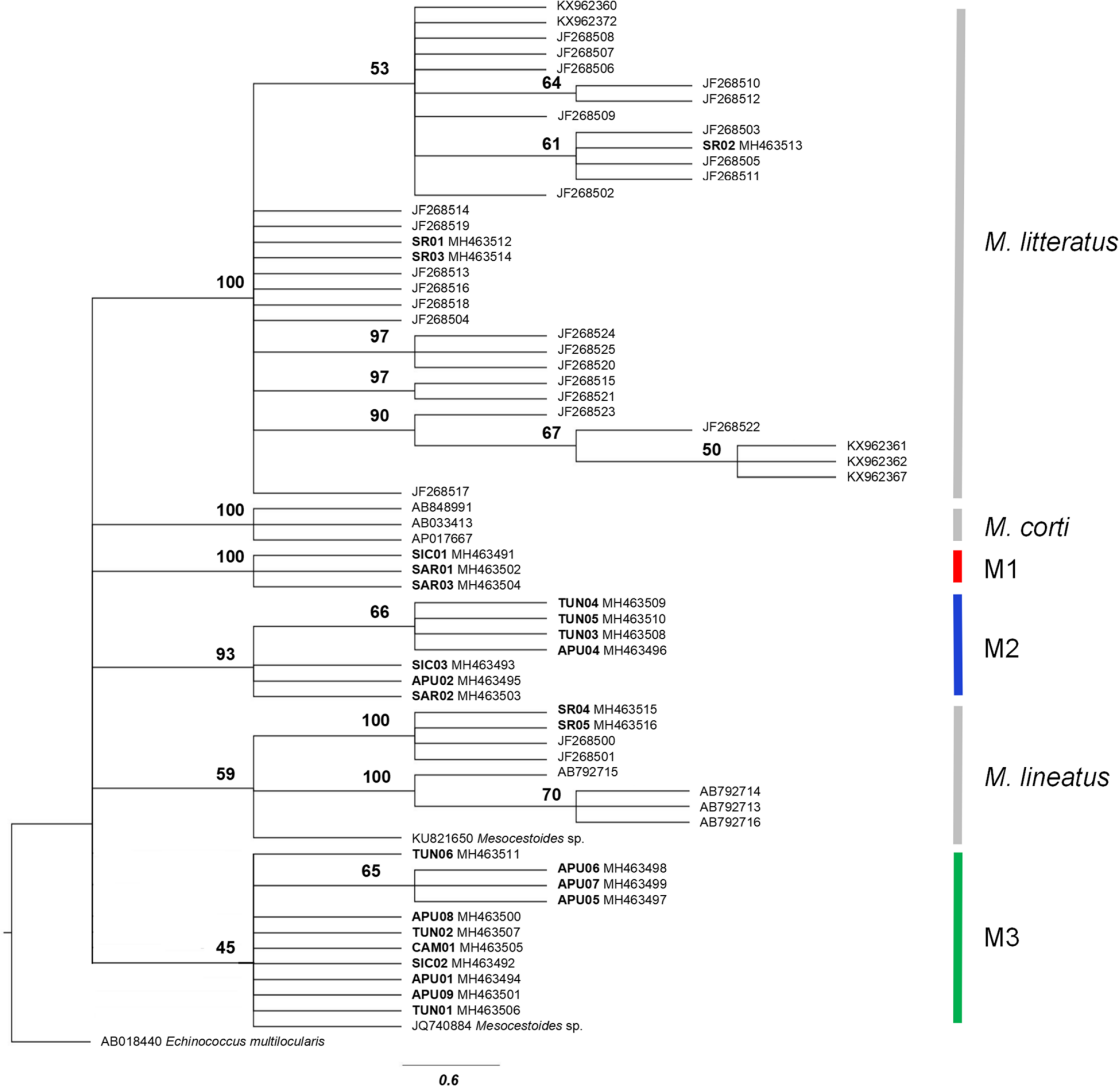
Maximum likelihood tree showing the interrelationships among *Mesocestoides* spp. based on the *cox*1 dataset. Outgroup: *Echinococcus multilocularis*. Only bootstrap support values > 45% are shown. The scale-bar indicates the number of substitutions per site. Sample codes are listed in Table 1. M1, M2, and M3 indicate the entities found by species delimitation analysis described in the text

The likelihood map based on *cox*1 gene dataset (see Additional file 3: Figure S1a) indicated a strong phylogenetic signal. The maximum likelihood (ML) tree analysis (Fig. 4) showed five supported and one unsupported (bootstrap value of 45%) clusters; three for *M. litteratus*, *M. corti/M. vogae* and *M. lineatus*, and the remaining for the Mediterranean *Mesocestoide*s spp. specimens analysed in the present study. Notably, the *M. lineatus* cluster included a GenBank sequence (KU821650) from Sicily. The individuals of *Mesocestoides* spp. examined here grouped into three different clusters (M1, M2 and M3 in Fig. 4). A consensus sequence of *Mesocestoides* sp., from GenBank (JQ740884) from Tuscany, Italy, was also included in the M3 group.

The species delimitation methods, Automatic Barcode Gap Discovery (ABGD) and Nucleotide Divergence Threshold (NDT) converged on the same results; for this reason, only the ABGD results are reported. Four entities were identified within the Mediterranean *Mesocestoides* spp. analysed. Overall, the composition of all the groups found by ABGD matched the clusters in the ML tree analysis. Evolutionary divergences were estimated between the ML clusters of *Mesocestoides* spp. (*M. litteratus*, *M.corti/M. vogae*, *M. lineatus*, M1, M2 and M3) (see Additional file 4: Table S3 for details)*.* Consistent with the previous analysis which converged in separating APU08 from the remaining samples, this specimen was considered as a further separate group to be tested.

**Fig. 5:**
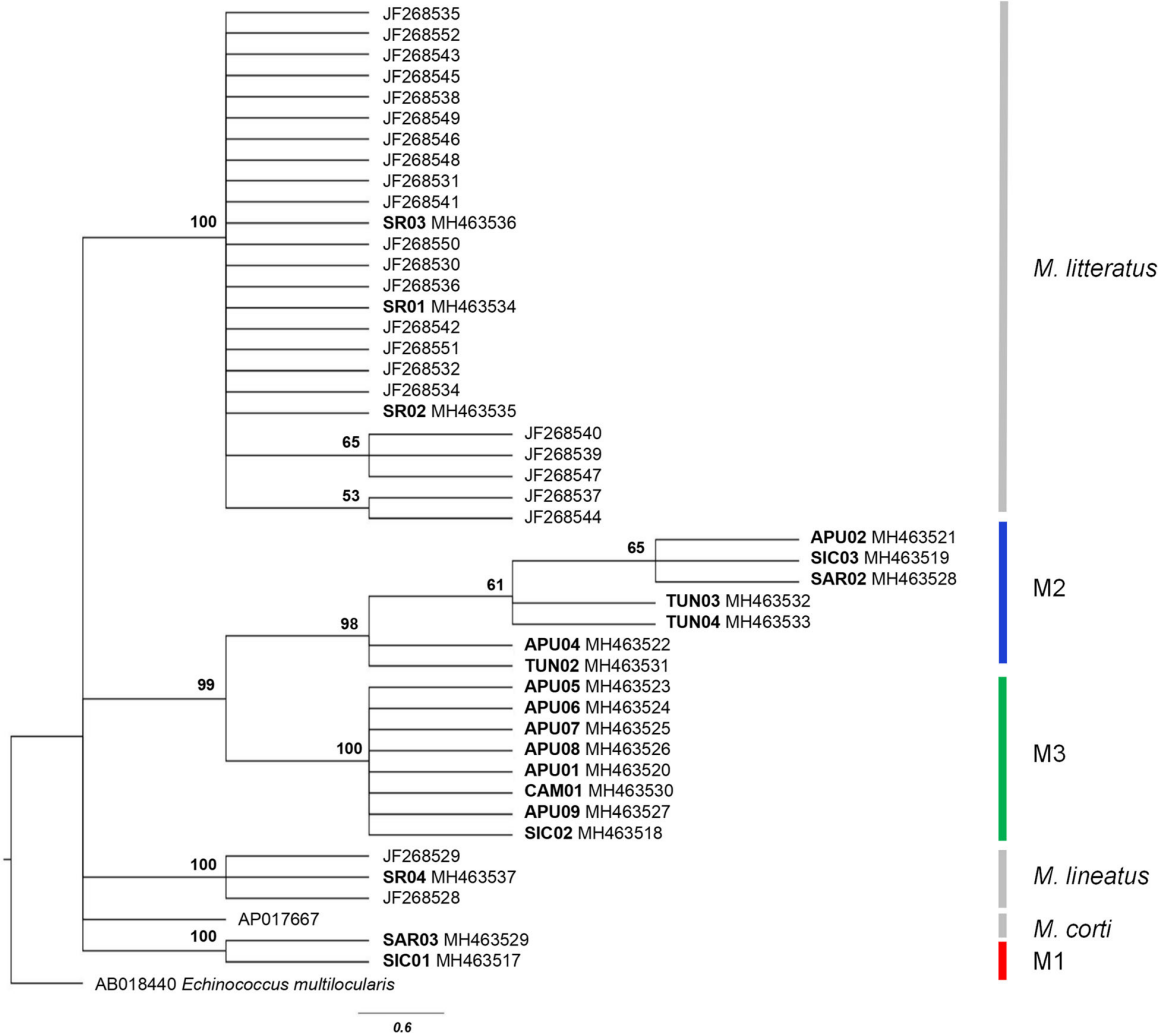
Maximum likelihood rooted tree showing the interrelationships among *Mesocestoides* spp. based on the *nad*1 dataset. Only bootstrap support values ≥ 50% are shown. The scale-bar indicates the number of substitutions per site. Sample codes are listed in Table 1. M1, M2 and M3 indicate the entities found by species delimitation analysis described in the text

The likelihood map based on the *nad*1 gene (see Additional file 3: Figure S1b) indicated a low phylogenetic signal. The ML tree analysis was consistent in showing the same results for the *cox*1 dataset (see Fig. 5 for a comparison). Additionally, the species delimitation methods (ABGD and NDT) converged on the same results, and the composition of the groups obtained exactly matched the clusters obtained by ML tree analysis. The ABGD method identified three entities within the Mediterranean *Mesocestoides* spp*.* analysed, with the position of the individual from Tunisia TUN02 representing the only discrepancy between the *cox*1 and *nad*1 ML tree and species delimitation analysis. Estimates of evolutionary divergences for *nad*1 gene are reported in the Additional file 5: Table S4.

## Discussion

This study on mitochondrial genetic variability of *Mesocestoides* spp. from domestic and wild carnivores in the Mediterranean area allowed us to gather a deep definition of the species delimitation among samples from southern Italy and Tunisia. Indeed, molecular analyses were consistent in pointing out three, well-defined, taxonomic *Mesocestoides* entities from Italy and Tunisia with high levels of genetic variation among individuals and no evidence of geographical structuring among entities (namely M1, M2 and M3 as in ML analysis). The occurrence of a sequence (GenBank: JQ740884) from Tuscany belonging to the M3 entity suggests that the range of distribution of these parasites probably extends north and not only confined to the Mediterranean region. Such a finding is consistent with a study [46], which highlighted the occurrence of a species genetically divergent from *M. lineatus*, *M. litteratus* and *M. corti*, in northern Italy. Two of the *Mesocestoides* entities in the present study (M2 and M3) displayed very low levels of genetic divergence among each other; the *nad*1 results evidenced the occurrence of a reciprocal monophyly between them, likely consistent with the presence of two *Mesocestoides* sister taxa in the Mediterranean area.

The statistical parsimony network analysis based on the *cox*1 dataset suggests that M2 and M3 probably originated in Tunisia, with an ancestor haplotype also present in Apulia. These findings could be consistent with the occurrence of early polymorphisms, maybe common to the southern Italy and Tunisia, possibly due to the translocation of domestic dogs since ancient times.

The levels of genetic divergence found between the Mediterranean *Mesocestoides* entities and *M. lineatus*, higher than those found between the *M. lineatus* geographical internal subgroups from Slovak Republic, Mongolia and Italy, further support the occurrence, at least in Italy and Tunisia, of *Mesocestoides* entities strongly divergent from *M. lineatus.* Notably the latter, which is described in the Italian Peninsula [2], was not identified in this work. Conversely, *M. lineatus* has been previously identified in Italy in a cat from Sicily [27], as evidenced by sequence GenBank: KU821650 included as an outlier within the *M. lineatus* cluster. This finding suggests that possibly several *Mesocestoides* spp. may occur in sympatry in southern Italy.

From a systematic perspective, the co-occurrence of different molecular entities, does not allow for unequivocal identification and description of the new taxa corresponding to the entities found. Furthermore, the inconsistency between morphological and molecular features supports the hypothesis that different environmental and ecological features interacted in the Mediterranean area to produce cryptic species within the genus *Mesocestoides* that are genetically divergent but morphologically indistinguishable from each other. In this context, it is important to underline the pivotal role of the molecular taxonomy, not only in identifying the cut-off to delimit species from each other, but also to the naming of the species itself, which result from the validation of the primary species hypotheses [47–53]. In fact, from a viewpoint that takes into account the appreciation of specific biodiversity, no species can be documented without a formal description, as well as no OTUs may substitute a species in any species checklist. [28, 54]. For this reason, the combined analysis of molecular and morphological data, in the light of the “integrative taxonomy approach” [55] will be used in the near future to provide both a satisfactory insight on the evolutionary processes and taxonomic richness, with a formal description of new species of the genus *Mesocestoides* in the Mediterranean area.

## Conclusions

The present study represents the first survey on mitochondrial genetic variability of *Mesocestoides* spp. from domestic and wild carnivores in the Mediterranean area that allowed to point out three defined, taxonomic *Mesocestoides* entities which are genetically divergent from *M. lineatus*, *M. litteratus* and *M. corti*.

## Additional files

Additional file 1: **Table S1.** Distribution (absolute frequencies) of *cox*1 haplotypes in 21 specimens from five Mediterranean sites. Sample codes are listed in Table 1. (DOCX 15 kb)

Additional file 2: **Table S2.** Distribution (absolute frequencies) of *nad*1 haplotypes in 17 specimens from five Mediterranean sites. Sample codes are listed in Table 1. (DOCX 15 kb)

Additional file 3: **Figure S1.** Likelihood mapping. **a** *cox*1 dataset. **b** *nad*1 dataset. For both panels: (i) distribution map of dots P, where P represents the likelihoods of the three possible unrooted trees for a set of four sequences (quartets) [54]. Dots close to the corners and to the sides represent tree-like and network-like phylogenetic signal. Central area represents star-like signal (phylogenetic noise); (ii) percentage distribution of the three possible unrooted trees; (iii) partitions of the area of the triangle into seven regions. The three trapezoids at the corners represent the areas supporting strictly bifurcating trees, that is the presence of a tree-like phylogenetic signal. The three rectangles on the sides represent regions where the decision between two trees is not obvious. The centre of the triangle represents sets of points P where all three trees are equally supported. (TIF 5240 kb)

Additional file 4: **Table S3.** Estimates of evolutionary divergence over sequence pairs between groups based on the *cox*1 dataset**.** Genetic distances, represented by the number of base substitutions per site from averaging over all sequence pairs between groups, are shown below the diagonal and standard deviations above the diagonal. Analyses were conducted using the K2P model. Sample codes are listed in Table 1. (DOCX 17 kb)

Additional file 5: **Table S4.** Estimates of evolutionary divergence over sequence pairs between groups based on the *nad*1 dataset. Genetic distances, represented by the number of base substitutions per site from averaging overall sequence pairs between groups, are shown below the diagonal and standard deviations are shown above the diagonal. Analyses were conducted using the K2P model. Sample codes are listed in Table 1. (DOCX 18 kb)

## Abbreviations

ML: Maximum likelihood
ABGD: Automatic Barcode Gap Discovery
NDT: Nucleotide Divergence Threshold
