# Supplementary material for: Species delimitation based on mtDNA genes suggests the occurrence of new species of Mesocestoides in the Mediterranean region
Source: Parasit Vectors. 2018 Dec 4;11:619. doi: 10.1186/s13071-018-3185-x (PMC6278086; doi:10.1186/s13071-018-3185-x)
Supplement: Supplementary file 1 — Table S1. Distribution (absolute frequencies) of cox1 haplotypes in 21 specimens from five Mediterranean sites. Sample codes are listed in Table 1. (DOCX 15 kb) [file 13071_2018_3185_MOESM1_ESM.docx]

**Additional file 1: Table S1.** Distribution (absolute frequencies) of *cox*1 haplotypes in 21 specimens from five Mediterranean sites. Sample codes are listed in Table 1

|  | **APU** | **CAM** | **SAR** | **SIC** | **TUN** | **Tot** | **GenBank ID** |
| --- | --- | --- | --- | --- | --- | --- | --- |
| **H1** |  |  |  | 1 |  | 1 | MH463491 |
| **H2** | 2 |  |  | 1 | 1 | 4 | MH463492 |
| **H3** | 1 |  | 1 | 1 |  | 3 | MH463493 |
| **H4** | 1 |  |  |  | 3 | 4 | MH463496 |
| **H5** | 3 |  |  |  |  | 3 | MH463497 |
| **H6** | 1 |  |  |  |  | 1 | MH463500 |
| **H7** |  |  | 1 |  |  | 1 | MH463502 |
| **H8** |  |  | 1 |  |  | 1 | MH463504 |
| **H9** |  | 1 |  |  |  | 1 | MH463505 |
| **H10** |  |  |  |  | 1 | 1 | MH463507 |
| **H11** |  |  |  |  | 1 | 1 | MH463511 |
