# Supplementary material for: Species delimitation based on mtDNA genes suggests the occurrence of new species of Mesocestoides in the Mediterranean region
Source: Parasit Vectors. 2018 Dec 4;11:619. doi: 10.1186/s13071-018-3185-x (PMC6278086; doi:10.1186/s13071-018-3185-x)
Supplement: Supplementary file 2 — Table S2. Distribution (absolute frequencies) of nad1 haplotypes in 17 specimens from five Mediterranean sites. Sample codes are listed in Table 1. (DOCX 15 kb) [file 13071_2018_3185_MOESM2_ESM.docx]

**Additional file 2: Table S2.** Distribution (absolute frequencies) of *nad*1 haplotypes in 17 specimens from five Mediterranean sites. Sample codes are listed in Table 1.

|  | **APU** | **CAM** | **SAR** | **SIC** | **TUN** | **Tot** | **GenBank ID** |
| --- | --- | --- | --- | --- | --- | --- | --- |
| **H1** |  |  |  | 1 |  | 1 | MH463517 |
| **H2** | 2 | 1 |  | 1 |  | 4 | MH463518 |
| **H3** | 1 |  | 1 | 1 |  | 3 | MH463519 |
| **H4** | 1 |  |  |  |  | 1 | MH463522 |
| **H5** | 4 |  |  |  |  | 4 | MH463523 |
| **H6** |  |  | 1 |  |  | 1 | MH463529 |
| **H7** |  |  |  |  | 1 | 1 | MH463531 |
| **H8** |  |  |  |  | 2 | 2 | MH463532 |
