# Supplementary figures and images for: Species delimitation based on mtDNA genes suggests the occurrence of new species of Mesocestoides in the Mediterranean region
Source: Parasit Vectors. 2018 Dec 4;11:619. doi: 10.1186/s13071-018-3185-x (PMC6278086; doi:10.1186/s13071-018-3185-x)

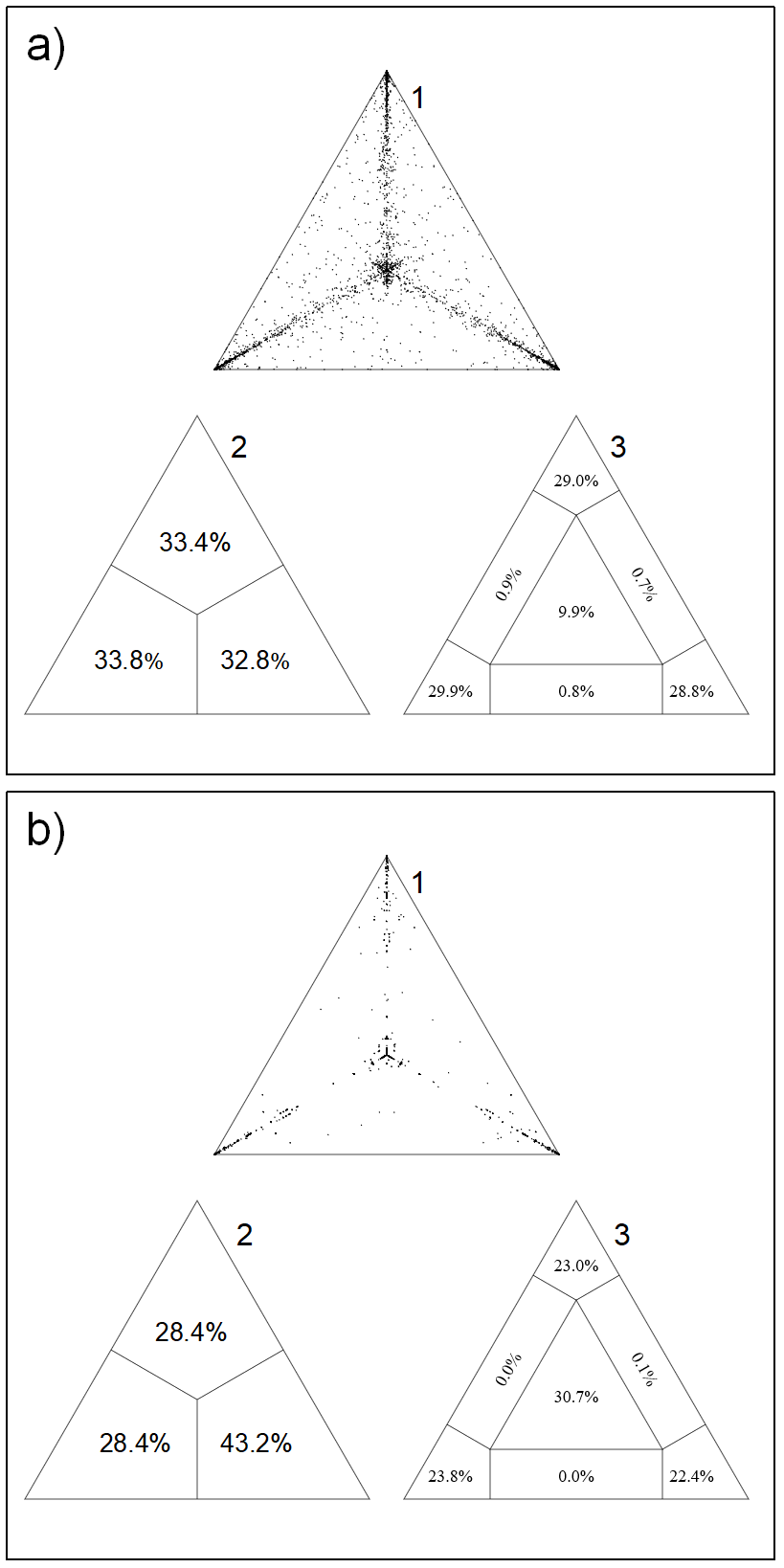

Supplement: Supplementary file 3 — Figure S1. Likelihood mapping. a cox1 dataset. b nad1 dataset. For both panels: (i) distribution map of dots P, where P represents the likelihoods of the three possible unrooted trees for a set of four sequences (quartets) [54]. Dots close to the corners and to the sides represent tree-like and network-like phylogenetic signal. Central area represents star-like signal (phylogenetic noise); (ii) percentage distribution of the three possible unrooted trees; (iii) partitions of the area of the triangle into seven regions. The three trapezoids at the corners represent the areas supporting strictly bifurcating trees, that is the presence of a tree-like phylogenetic signal. The three rectangles on the sides represent regions where the decision between two trees is not obvious. The centre of the triangle represents sets of points P where all three trees are equally supported. (TIF 5240 kb) [file 13071_2018_3185_MOESM3_ESM.tif]
