# Supplementary material for: Species delimitation based on mtDNA genes suggests the occurrence of new species of Mesocestoides in the Mediterranean region
Source: Parasit Vectors. 2018 Dec 4;11:619. doi: 10.1186/s13071-018-3185-x (PMC6278086; doi:10.1186/s13071-018-3185-x)
Supplement: Supplementary file 5 — Table S4. Estimates of evolutionary divergence over sequence pairs between groups based on the nad1 dataset. Genetic distances, represented by the number of base substitutions per site from averaging overall sequence pairs between groups, are shown below the diagonal and standard deviations are shown above the diagonal. Analyses were conducted using the K2P model. Sample codes are listed in Table 1. (DOCX 18 kb) [file 13071_2018_3185_MOESM5_ESM.docx]

**Additional file 5: Table S4.** Estimates of evolutionary divergence over sequence pairs between groups based on the *nad1* dataset. Genetic distances, represented by the number of base substitutions per site from averaging over all sequence pairs between groups, are shown below the diagonal and standard deviations above the diagonal. Analyses were conducted using the K2P model. Sample codes are listed in Table 1.

|  | **M1** | **M2** | **M3** | **M. lineatus** | **M. litteratus** | **M. corti** | **E. multilocularis** |
| --- | --- | --- | --- | --- | --- | --- | --- |
| **M1** |  | *0.031* | *0.032* | *0.026* | *0.027* | *0.026* | *0.039* |
| **M2** | 0.158 |  | *0.016* | *0.029* | *0.034* | *0.031* | *0.043* |
| **M3** | 0.165 | 0.053 |  | *0.031* | *0.031* | *0.033* | *0.042* |
| 1. ***lineatus*** | 0.154 | 0.150 | 0.160 |  | *0.033* | *0.030* | *0.046* |
| ***M. litteratus*** | 0.143 | 0.192 | 0.165 | 0.178 |  | *0.032* | *0.038* |
| 1. ***corti*** | 0.121 | 0.165 | 0.179 | 0.172 | 0.180 |  | *0.041* |
| 1. ***multilocularis*** | 0.251 | 0.286 | 0.273 | 0.313 | 0.245 | 0.258 |  |
